# Simultaneous exposure to nitrate and low pH reduces the blood oxygen-carrying capacity and functional performance of a freshwater fish

**DOI:** 10.1093/conphys/coz092

**Published:** 2020-01-23

**Authors:** Daniel F Gomez Isaza, Rebecca L Cramp, Craig E Franklin

**Affiliations:** School of Biological Science, The University of Queensland, Brisbane, Queensland 4072, Australia

**Keywords:** Aerobic scope, blood, blood oxygen affinity, ecotoxicology, methaemoglobin, swimming performance

## Abstract

Human activities present aquatic species with numerous of environmental challenges, including excessive nutrient pollution (nitrate) and altered pH regimes (freshwater acidification). In isolation, elevated nitrate and acidic pH can lower the blood oxygen-carrying capacity of aquatic species and cause corresponding declines in key functional performance traits such as growth and locomotor capacity. These factors may pose considerable physiological challenges to organisms but little is known about their combined effects. To characterise the energetic and physiological consequences of simultaneous exposure to nitrate and low pH, we exposed spangled perch (*Leiopotherapon unicolor*) to a combination of nitrate (0, 50 or 100 mg L^−1^) and pH (pH 7.0 or 4.0) treatments in a factorial experimental design. Blood oxygen-carrying capacity (haemoglobin concentration, methaemoglobin concentrations and oxygen equilibrium curves), aerobic scope and functional performance traits (growth, swimming performance and post-exercise recovery) were assessed after 28 days of exposure. The oxygen-carrying capacity of fish exposed to elevated nitrate (50 and 100 mg L^−1^) was compromised due to reductions in haematocrit, functional haemoglobin levels and a 3-fold increase in methaemoglobin concentrations. Oxygen uptake was also impeded due to a right shift in oxygen–haemoglobin binding curves of fish exposed to nitrate and pH 4.0 simultaneously. A reduced blood oxygen-carrying capacity translated to a lowered aerobic scope, and the functional performance of fish (growth and swimming performance and increased post-exercise recovery times) was compromised by the combined effects of nitrate and low pH. These results highlight the impacts on aquatic organisms living in environments threatened by excessive nitrate and acidic pH conditions.

## Teaser

Exposure to elevated nitrate and acidic pH impaired oxygen uptake and reduced swimming performance, growth and post-exercise recovery in juvenile spangled perch. Reductions in performance were caused by lowered blood oxygen-carrying capacity, whereby haemoglobin, haematocrit and oxygen binding were compromised by nitrate and low pH.

## Introduction

The acceleration of anthropogenic activity has caused novel or extreme environmental challenges for species to navigate. As a result, freshwater species must now contend with complex combinations of environmental threats, which include habitat degradation, pollution and climate change (Dudgeon *et al.*, 2006; Collen *et al.*, 2014, ). Further pressing is the lack of available data on how species cope when confronted with multiple environmental challenges simultaneously (Reid *et al.*, 2019). It is clear that stressor interactions are often non-linear (Jackson *et al.*, 2016), meaning that predictability of single-stressor studies are likely to over- or under-estimate species responses to environmental change. Robust experimental assessments of species responses to multiple environmental threats are necessary to understand the physiological constraints beyond which performance and survival are at risk (McKenzie *et al.*, 2016).

Nutrient pollution (i.e. nitrate and phosphorous P inputs) is regarded as one of the most common and widespread forms of habitat degradation (Fowler *et al.*, 2013). Anthropogenic activities have caused a rampant increase in nitrate concentrations, leading from fertilizer use, sewage and industrial runoff and the release of nitrogen from aquaculture operations (Petzoldt and Uhlmann, 2006). Such increases mean that aquatic species risk regular exposure to unnaturally high levels of nitrate. As a biotoxin (i.e. a toxin of biological origin), elevated nitrate has received little attention. Research to date has centred on determining the lethal concentrations of nitrate (e.g. LC_50_) (Adelman *et al.*, 2009; Learmonth and Carvalho, 2015; Lou *et al.*, 2016), and regulatory agencies have developed guidelines for ‘safe’ nitrate levels based on limited datasets (Environment Australia, 2000; Canadian Council of Ministers of the Environment, 2012; European Commission, 2018). Moreover, the sub-lethal effects of nitrate have been overlooked by environmental agencies despite evidence pointing towards reduced growth (McGurk *et al.*, 2006; van Bussel *et al.*, 2012; Monsees *et al.*, 2017) and activity levels (McGurk *et al.*, 2006; Davidson *et al.*, 2014), slowed development (Smith and Burgett, 2012; Wang *et al.*, 2015) and reduced fecundity (Alonzo and Camargo, 2013; Kellock *et al.*, 2018) in fish and amphibians chronically exposed to elevated levels of nitrate. The determination of lethal concentrations are, therefore, of little ecological relevance as sub-lethal reductions in performance will likely be the proximate cause of mortality in disturbed ecosystems (Connon *et al.*, 2012). Instead, the measurements of complex physiological traits, including aerobic scope (i.e. maximum-standard oxygen uptake) and exercise performance, may be more reliable indicators of sub-lethal toxicological effects (McKenzie *et al.*, 2007).

The measurement of aerobic scope has become a useful physiological framework to integrate the effects of various environmental stressors and predicts the amount of residual energy available for key functional performance traits such as locomotor capacity, growth and foraging activity (Claireaux and Lefrançois, 2007; Sokolova, 2013). For instance, exposure to nitrite caused ~40% decrease in aerobic scope and translated to poorer sustained swimming capacity in striped catfish (*Pangasianodon hypophthalmus*) (Lefevre *et al.*, 2011). Similarly, declines in aerobic scope following exposure to mild hypoxia corresponded with a reduced growth rate in Atlantic cod (*Gadus morhua*) (Chabot and Dutil, 1999). Exposure to elevated nitrate levels has been shown to reduce aerobic scope in crayfish (Gomez Isaza *et al.*, 2018) and cause declines in functional performance traits in fish (McGurk *et al.*, 2006; van Bussel *et al.*, 2012; Davidson *et al.*, 2014), yet the mechanisms underlying a reduced aerobic capacity following nitrate exposure remain unclear.

Nitrate is thought to impact on the blood oxygen-carrying capacity of aquatic species due to the formation of methaemoglobin. Nitrate enters the body via passive diffusion across the gills and results in the endogenous conversion of nitrate to nitrite (Camargo *et al.*, 2005; Monsees *et al.*, 2017), which then causes methaemoglobin formation. Methaemoglobin is a haemoglobin isoform that is unable to reversibly bind oxygen and causes an inherent loss of oxygen transport capacity (i.e. functional anaemia; Jensen, 2003; Avilez *et al.*, 2004). Under normal conditions, methaemoglobin circulates at low levels (<5%) within the body (Williams *et al.*, 1993). Signs of methaemoglobinaemia appear as levels exceed 10% methaemoglobin or more, although some fish are relatively tolerant of elevated methaemoglobin concentrations (Lefevre *et al.*, 2011; van Bussel *et al.*, 2012). Exposure to nitrite/nitrate can also cause other haematological disturbances, including decreases in total haemoglobin (Grabda *et al.*, 1974; Avilez *et al.*, 2004; Monsees *et al.*, 2017; Yang *et al.*, 2019) and haematocrit (Monsees *et al.*, 2017), and reduce oxygen binding affinity of functional haemoglobin (Jensen *et al.*, 1987; Jensen, 1990). However, evidence for nitrate-induced methaemoglobinaemia is scarce and requires experimental validation.

In addition to nitrate pollution, species inhabiting developing waterways likely face other environmental challenges that can mediate the toxicity of nitrate. The potential interactions between nitrate and other environmental stressors are not well understood, but recent studies indicate that such interactions may be quite complex (Hatch and Blaustein, 2000; Ortiz-Santaliestra *et al.*, 2010; Smith *et al.*, 2013; Gomez Isaza *et al.*, 2018). Of particular threat are environmental stressors that can enhance nitrate toxicity either by increasing its uptake into the body and/or by having a combined impact on blood oxygen-carrying capacity. Environmental pH is one factor that may modify the toxicity of nitrate to aquatic organisms. In freshwater, acute exposure to low pH (less than pH 5.0) increases the loss of electrolytes (Na^+^ and Cl^−^) across the gills (McDonald, 1983). A loss of electrolytes reduces plasma volume, increasing blood viscosity (i.e. increased haematocrit), and causes haemoglobin dysfunctions (Milliguin and Wood, 1982; Witters *et al.*, 1990), which, together, limit oxygen transport. Depending on water calcium levels, acid-base disturbances can also arise following low pH exposure to cause extracellular acidosis (Wood and McDonald, 1982), which lowers haemoglobin–oxygen binding affinity (Rummer *et al.,* 2013). The effects of low pH manifest to impair a suite of whole animal physiological traits, including reduced growth (Ye *et al.,* 1991; Dockray *et al.,* 1996), locomotor performance (Ye *et al.,* 1991; Butler *et al.,* 1992) and increased maintenance metabolic costs (Ye *et al.,* 1991; Butler *et al.,* 1992). Damage to paracellular junctions (Freda *et al.*, 1991) may also arise as a result of low pH exposure, which facilitates the uptake and accumulation of pollutants (Çoĝun and Kargin, 2004).

The influence of environmental pH on nitrate toxicity is relatively unknown. However, nitrite toxicity, which has a similar toxic mechanism to nitrate, is exacerbated by extreme low pH exposure (i.e. at pH values outside the normal adaptive range of a species). Experiments on nitrite-exposed fish have reported strong haematological effects (elevated methaemoglobin, nitrite concentrations) when exposed simultaneously to low pH (Meade and Perrone, 1980). Meade and Perrone (1980) proposed that at low pH, uptake characteristics of ion transporter in the gills are altered, which enhance nitrite toxicity. A similar effect is expected in fish exposed simultaneously to elevated nitrate and low pH. Therefore, it is hypothesized that elevated nitrate and low pH will combine to hamper the oxygen-carrying capacity of fish and cause cascading declines of key physiological traits.

The present study aimed to characterize the interaction between nitrate and low pH exposure on the blood oxygen-carrying capacity and functional performance of a freshwater fish species. Specifically, we focussed on the spangled perch (*Leiopotherapon unicolor*), a freshwater fish found throughout Australian (e.g. the Murray–Darling rivers) systems. Nutrient effluent is the main driver of poor water quality in the Murray–Darling basin (Thorburn *et al.*, 2003; Hamer *et al.*, 2004), with nitrate concentrations frequently exceeding water nitrate safety guidelines (50 mg L^−1^) (Environment Australia, 2000). Further, acidification of freshwaters has occurred in parts of Australia’s eastern coastline due to the presence of acid sulphate soils (Baldwin, 2011; Kilminster and Cartwright, 2011). Acid sulphate soils can produce highly acidic discharges (pH < 4.0; Sammut *et al.*, 1994) and can cause mass mortality of fish and crustaceans (Sammut *et al.*, 1996; White *et al.*, 1997; Russell *et al.*, 2011).

To test the interactive effects of nitrate and low pH, we employed a fully factorial experimental design and exposed juvenile spangled perch to two pH levels (either 7.0 or 4.0) and three nitrate concentrations (0, 50 and 100 mg L^−1^${\mathrm{NO}}_3^{-}$) for 4 weeks prior to blood sampling and performance testing. The interactive effects of elevated nitrate and low pH on blood oxygen-carrying capacity [haemoglobin and methaemoglobin concentrations, haematocrit and oxygen equilibrium curves (OECs)] were measured. Further, aerobic scope, growth rate, swimming performance and post-exercise recovery time were measured. We predicted that chronic exposure to low pH and elevated nitrate would increase nitrate accumulation in the plasma and cause synergistic reductions to the blood oxygen-carrying capacity and aerobic performance of juvenile spangled perch.

## Material and methods

### Experimental animals

Three hundred juvenile spangled perch (*L. unicolor*; mean ± SD: mass, 9.42 ± 2.78 g and total length, 8.50 ± 0.78 cm) were sourced from a commercial hatchery (Australian Native Fish Enterprises, Kallangur, Australia) and transported to The University of Queensland. Fish were equally distributed between eighteen 40-L glass tanks (60 × 29 × 24, L × W × H) at a density of 17 fish per tank. Each tank was equipped with a sponge filter for filtration. Tanks were filled with filtered tap water (Na^+^ = 1.9, Ca^2+^ = 0.6, Cl^−^ = 1.1, K^+^ = 0.1 mmol L^−1^) and held at 21.5 ± 1°C under a 12:12-h light–dark cycle. Fish were fed daily with sinking pellets (0.2 mm Ridley Aqua-feeds, Melbourne, Australia) and bloodworms (Orca, Nijimi Pty Ltd, Sydney, Australia) and maintained under these conditions for 1 month prior to experimentation. All experiments were performed in compliance with The University of Queensland animal ethics requirements (permit number SBS/249/17).

### Experimental design

We employed a full 2 × 3 factorial design with two pH levels (7.0 and 4.0) and three nitrate concentrations (0, 50 and 100 mg L^−1^${\mathrm{NO}}_3^{-}$). Each factorial combination was replicated three times at the tank level. Nitrate concentrations were prepared using reagent-grade sodium nitrate (ThermoFisher Scientific, Scoresby, Australia) and measured spectrophotometrically as described by Edwards *et al.* (2006)**.** Water pH was adjusted by adding dilute (10%) sulfuric acid and monitored using a handheld digital pH meter (LAQUA Compact pH meter, Horiba Scientific). Partial water changes (80–90%) were made on alternate days to maintain experimental conditions. Water quality parameters were measured once daily over the course of the experiment and are displayed in Table 1. Treatment pH and nitrate levels did not deviate from nominal concentrations by >0.14 pH units or by 15 mg L^−1^${\mathrm{NO}}_3^{-}$, respectively. Fish were exposed to experimental treatments for 4 weeks prior to performance testing. Food was withheld for 24 h prior to all experiments to ensure fish were in a post-absorptive state.

**Table 1:** Water quality parameters (ammonia, nitrite, nitrate and pH) over the 4-week exposure period

Treatment (pH/nitrate)	Ammonia (mg L^−1^)	Nitrite (mg L^−1^)	Nitrate (mg L^−1^)	pH	Temperature (°C)
7/0	0.04 (± 0.02)	0.11 (± 0.02)	5.31 (± 0.34)	6.97 (± 0.01)	21.76 (± 0.05)	
7/50	0.03 (± 0.04)	0.12 (± 0.02)	55.12 (± 0.85	6.97 (± 0.01)	21.77 (± 0.02)	
7/100	0.02 (± 0.03)	0.13 (± 0.01)	99.91 (± 0.73)	6.97 (± 0.00)	21.79 (± 0.10)	
4/0	0.18 (± 0.04)	0.0 (± 0.0)	2.08 (± 0.21)	3.95 (± 0.01)	21.91 (± 0.06)	
4/50	0.19 (± 0.04)	0.0 (± 0.0)	51.41 (± 1.77)	3.95 (± 0.01)	21.96 (± 0.01)	
4/100	0.21 (± 0.03)	0.0 (± 0.01)	97.51 (± 1.31)	3.95 (± 0.00)	21.97 (± 0.05)	

Data are presented as mean (± standard deviation).

### Growth rates

The body mass (wet mass, g) and total length (cm) of all fish was measured at the start (M_I_) and after (M_F_) 4 weeks of exposure to experimental treatments, and tank averages were calculated. Fish were individually weighed using an electronic balance (Kern KB1200-2 N, Balingen, Germany), measured and immediately returned to their holding tanks. Absolute growth rates (AGR, g d^−1^) were calculated as the absolute change in mass overtime (AGR = M_F_ − M_I_/days) (Lugert *et al.*, 2014). Fish condition factor (K) was calculated as K = (mass/length^3^) × 100. Scaled mass index (SMI) was also calculated as an index of body condition following the instructions of Peig and Green (2009); it was calculated as SMI = *W*_i_ [*L*_0_/*L*i]b^SMA^ where *W*_i_ and *L*_i_ are the mass and length of each fish, respectively; *L*_o_ is an arbitrary value of *L*, here chosen as the arithmetic mean of the length of our study population (8.57 cm); and b^SMA^ is the scaling exponent from the slope of a standardized major axis (SMA) regression of the mass–length relationship. We calculated b^SMA^ scaling exponent for our species was 3.06.

### Swimming performance

Swimming performance trials were conducted in a 10-L flow-controlled hydraulic flume (Loligo, Tjele, Denmark; swimming chamber dimensions = 40 × 10 × 10 cm, L × W × H). Water speeds were calibrated using a Prandtl-pitot tube, as described by Kern *et al*. (2018). Water in the flume was constantly aerated, and temperature was maintained at 21.5 ± 1°C using a 300-W water heater (Eheim, Stuttgard, Germany). Water parameters in the flume (pH and nitrate) were adjusted prior to fish introduction to reflect fish treatment group. Fish (7 fish per treatment) were netted from their holding tanks and individually placed in the swimming chamber of the flume. The anterior portion of the flume was covered with black plastic to encourage the fish to stay in the anterior part of the swim chamber. Fish were allowed to habituate to flume conditions for at least 1 h at a water velocity of 0.05 m s^−1^. Swimming performance was assessed as the critical swimming speed (*U*_CRIT_), which began at a water velocity of 0.2 m s^−1^ (~1.5–2 body lengths per second; BL s^−1^) and progressively increased every 5 min at a rate of 0.03 m s^−1^ until the fish fatigued. Total swimming time and water velocity at fatigue were recorded to calculate *U*_CRIT_ using Brett’s (1964) equation:

$$ {U}_{\mathrm{CRIT}}={U}_F+\left(\frac{T_F}{T_I}\right){U}_I, $$

where *U_F_* is the highest water velocity maintained for the entire 5-min interval (m s^−1^), *U_I_* is the water velocity increment (0.03 m s^−1^), *T_F_* is the time swum during the final increment (s) and *T_I_* is an entire velocity interval (300 s). Swimming performance was standardized for body length and expressed as BL s^−1^. The cross-sectional body area of the fish did not exceed 10% of the cross-sectional area of the swimming chamber, therefore corrections for solid blocking effects were not necessary (Bell and Terhune, 1970).

### Oxygen uptake and excess post-exercise oxygen consumption

Oxygen uptake rate ($\dot{\mathrm{M}}$O_2_) of fish was measured using intermittent-flow through respirometry (Clark *et al.*, 2013). Two acrylic respirometers (693 mL total volume including tubing) were individually submerged in black, 96-L tanks filled with filtered freshwater water at the appropriate pH and nitrate concentrations for each treatment. Each respirometer was fitted with two circulation loops. The first loop was fitted with a continuously operating water pump (Eheim 1048–219, Germany), which circulated the water within the respirometer and past an oxygen flow-through cell (Presens, Regensburg, Germany). A fibre-optic cable connected to a Fibox 3 reader (Presens, Regensburg, Germany) was fixed to the oxygen flow-through cell and measured oxygen concentrations within the respirometers every second. A second circulation loop comprised a pump connected to an automated timer, which flushed the respirometers with oxygenated water from the surrounding water bath. Timers were set on a 15-min on/off cycle and ensured that oxygen saturation did not drop below 75% during all trials. The water baths were continuously aerated using air stones and temperature within the water bath was maintained at 21.5 ± 1 C using a chiller/heater (Teco TK1000, USA).

Seven spangled perch from each treatment were randomly selected from their holding tanks and individually placed inside respirometer chambers. Fish were introduced to respirometry chambers prior to the first $\dot{\mathrm{M}}$O_2_ recording at ~17:00 and remained inside the respirometers for at least 14 h as oxygen saturation within the respirometers was continuously measured. Fish $\dot{\mathrm{M}}$O_2_ (mg O_2_ kg^−1^ h^−1^) was calculated as the slope of the decline in oxygen concentration inside the respirometers during the closed phase of the respirometry cycles. Specifically, $\dot{\mathrm{M}}$O_2_ was calculated as follows:

$$ \dot{\mathrm{M}}{\mathrm{O}}_2=\Delta {\mathrm{O}}_2/\Delta t\times \mathrm{V}, $$

where $\Delta$O_2_ is the rate of change of oxygen concentration inside the respirometer containing a fish, $\Delta$*t* is the change in time over which the $\Delta$O_2_ was measured and *V* is the volume of the respirometer minus the volume of the fish (assuming 1 g displaces 1 mL of water). To account for bacterial respiration, background oxygen consumption was measured after each trial and subtracted from $\dot{\mathrm{M}}$O_2_ measurements. Standard oxygen uptake ($\dot{\mathrm{M}}$O_2STANDARD_) was determined as the mean of the lowest 10% of $\dot{\mathrm{M}}$O_2_ values during the entire measurement period (Clark *et al.*, 2013). To obtain maximum oxygen uptake ($\dot{\mathrm{M}}$O_2MAX_), fish were removed from respirometers, swum to exhaustion following the *U*_CRIT_ protocol and then returned to their respirometers. We opted to measure $\dot{\mathrm{M}}$O_2MAX_ following exhaustive exercise rather than after a chase protocol (e.g. 3-min chase, 1-min air exposure) because preliminary data showed that prolonged swimming in a swim tunnel elicited a greater rate of oxygen uptake than the chase protocol in spangled perch. Fish were returned to respirometers and oxygen uptake was measured at 1, 2, 3, 4, 5, 10, 30, 60, 90 and 120 min after exhaustive exercise. $\dot{\mathrm{M}}$O_2MAX_ was measured as the greatest decline in oxygen over a 1-min period. Excess post-exercise oxygen consumption (EPOC; mL O_2_ kg^−1^ h^−1^) was estimated for each individual by calculating the area under the exponential recovery curve until the oxygen uptake rate returned to with 120% of $\dot{\mathrm{M}}$O_2STANDARD_ (Fu *et al.*, 2007). EPOC duration (h) was calculated as the time from exercise to when $\dot{\mathrm{M}}$O_2_ returned to standard levels. Absolute aerobic scope (AAS = $\dot{\mathrm{M}}$O_2MAX_ $-\dot{\mathrm{M}}$O_2STANDARD_) and factorial aerobic scope (FAS = $\dot{\mathrm{M}}$O_2MAX_/$\dot{\mathrm{M}}$O_2STANDARD_) were also calculated.

### Blood collection and analysis

Blood was sampled terminally by severing the caudal peduncle and collecting blood directly into three heparinized haematocrit tubes. Two of the haematocrit tubes were centrifuged (micro-haematocrit centrifuge; Hawksley, Sussex, UK) for 3 min at 5000 *g* and haematocrit (H_CT_) was measured as the proportion of red blood cells in whole blood. Blood from the remaining haematocrit tube was aliquoted into two 1.5 mL Eppendorf tubes for haemoglobin and methaemoglobin concentration determination. Haemoglobin concentration ([H_B_]) was determined spectrophotometrically at 405 nm and quantified against a standard curve of known [H_B_] using a Sigma-Aldrich haemoglobin assay kit (MAK115; St Louis, USA). Mean corpuscular haemoglobin concentration (MCHC) was calculated as [H_B_] × 10/H_CT_. Methaemoglobin concentration was determined by diluting 20 μL of whole blood in 1 mL of 34-mM phosphate buffer at pH 7.3. The haemolysate was centrifuged (microfuge^®^18 centrifuge, Beckman Coulter, Brea, USA) for 3 min at 12 000 *g,* and the absorbance (DU800 spectrophotometer, Beckman Coulter, Brea, USA) was measured at 560, 576 and 630 nm, following published protocols (Benesch *et al.*, 1973). Plasma N${\mathrm{O}}_3^{-}$ and N${\mathrm{O}}_2^{-}$ were quantified using a colorimetric assay kit (kit number 780001; Cayman Chemicals, Ann Arbor, USA) following the manufacturer’s instructions. All assays were run in duplicate.

### Oxygen equilibrium curves

OECs were determined using a Hemox-Analyser (Model B, TCS Scientific Corp., USA). A sample of 50 μL of blood was suspended in 5 mL of buffered saline (Hemox™-Solution, pH 7.4), 20 μL of bovine serum albumin (BSA, additive A, Hemox™) and 10 μL of an anti-foaming agent (additive B, Hemox™). Samples were analysed at 22°C. Zero percent saturation haemoglobin oxygen was achieved by bubbling samples using 100% nitrogen (compressed nitrogen pure, gas code 032, BOC, North Ryde, Australia), and then air (i.e. 20.9% oxygen, compressed air gas code 054) was used to obtain full saturation. OECs and *P*_50_ values were plotted and obtained by the Hemox Analyser software (Hemox analytical software version 2, TCS Scientific Corp.). The PO_2_ values were obtained for 0, 10, 20, 30, 40, 50, 60, 70, 80, 90, 100% oxygenation of H_B_ for each blood sample and the mean from each treatment group was used to create mean OECs for each treatment.

### Statistical analysis

Statistical analyses were performed in the R programming environment (https://www.r-project.org/) using the RStudio interface (version 1.0.153). All variables were tested for normality and homoscedasticity with Shapiro-Wilk and Levene tests, respectively. Swimming performance and methaemoglobin concentration data were log-transformed due to violations of normality. Linear mixed effects models were used to test for statistical differences between nitrate and pH treatments for each of the following response variables: blood (plasma${\mathrm{NO}}_2^{-}$, plasma${\mathrm{NO}}_3^{-}$, H_CT_, [H_B_], MetH_B_, MCHC and *P*_50_), growth, *U*_CRIT_, $\dot{\mathrm{M}}$O_2STANDARD,_$\dot{\mathrm{M}}$O_2MAX_, AAS and FAS. Nitrate and pH were included as fixed effects in all models , and tank was included as random effects. Body mass was included as a covariate in all analyses, except for the *U*_CRIT_ analysis where total length was included as a covariate. The *lme* function of the *nlme* package (Pinheiro *et al.*, 2017) was used for all the aforementioned analyses. In case of statistically significant differences, *post hoc* pairwise comparisons were performed using the *lsmeans* function of the *lsmeans* package (Lenth, 2016). A censored regression analysis was used to model EPOC duration as a function of pH, nitrate and body mass using the *vglm* function of the *VGAM* package (Yee, 2018). A censored regression analysis was used for EPOC duration as all fish had not returned to within 120% $\dot{\mathrm{M}}$O_2STANDARD_ within the 2-h recovery period. Significant differences were accepted as *P* < 0.05. Data are presented as mean ± standard error unless otherwise stated.

## Results

### Fish growth and condition

Independent exposure to elevated nitrate (*F*_2,12_ = 8.16, *P* = 0.006) or low pH (*F*_1,12_ = 3.69, *P* = 0.01) had strong, depressive effects on fish growth. Indeed, fish exposed to elevated nitrate (50 or 100 mg L^−1^) or pH 4.0 experienced marginal growth rates or lost mass over the 28-day growth experiment (Table 2). When combined, there was a significant antagonistic interaction (i.e. less than the two stressors independently) between nitrate and pH treatment on the absolute fish growth rate of fish (*F*_2,12_ = 9.01, *P* = 0.004; Table 2). Similarly, the body condition (Fulton’s K) of fish was significantly affected independently by nitrate (*F*_2,12_ = 7.35, *P =* 0.008), pH (*F*_1,12_ = 7.35, *P =* 0.003) and marginally by their interaction (*F*_2,12_ = 3.86, *P =* 0.05). *Post hoc* pairwise comparisons showed that condition factor was lowest in fish exposed to combined elevated nitrate and low pH (Table 2). Comparable results were seen when fish condition was calculated as the SMI and survival was high in all treatments (Table 2).

**Table 2:** Fish initial and final masses, Fulton’s condition factor, SMI and survival

	pH 4.0			pH 7.0		
	0	50	100	0	50	100
Initial mass	9.46 (± 0.66)^A^	10.50 (± 1.36)^A^	9.32 (± 0.61)^A^	9.48 (± 1.46)^A^	8.89 (± 0.51)^A^	9.16 (± 0.85)^A^
Final mass	9.50 (± 0.74)^A^	10.54 (± 1.06)^A^	9.38 (± 0.65)^A^	9.85 (± 1.52)^A^	8.87 (± 0.50)^A^	9.24 (± 0.73)^A^
Growth	0.03 (± 0.05)^B^	0.04 (± 0.01)^B^	0.05 (± 0.04)^B^	0.36 (± 0.05)^A^	−0.05 (± 0.02)^B^	0.07 (± 0.09)^B^
Condition	1.48 (± 0.01)^B^	1.44 (± 0.02)^BC^	1.42 (± 0.02)^C^	1.52 (± 0.01)^A^	1.46 (± 0.02)^BC^	1.48 (± 0.03)^B^
SMI	9.30 (± 0.02)^B^	9.09 (± 0.08)^BC^	8.93 (± 0.09)^C^	9.61 (± 0.04)^A^	9.17(± 0.06)^BC^	9.32 (± 0.11)^B^
Survival (%)	100 (± 0)^A^	100 (± 0)^A^	91.6 (± 4.81)^A^	100 (± 0)^A^	97.9 (± 1.20)^A^	100 (± 0)^A^

Superscripted uppercase letters represent statistical difference between treatment groups and data are presented as mean (± standard deviation).

**Figure 1:**
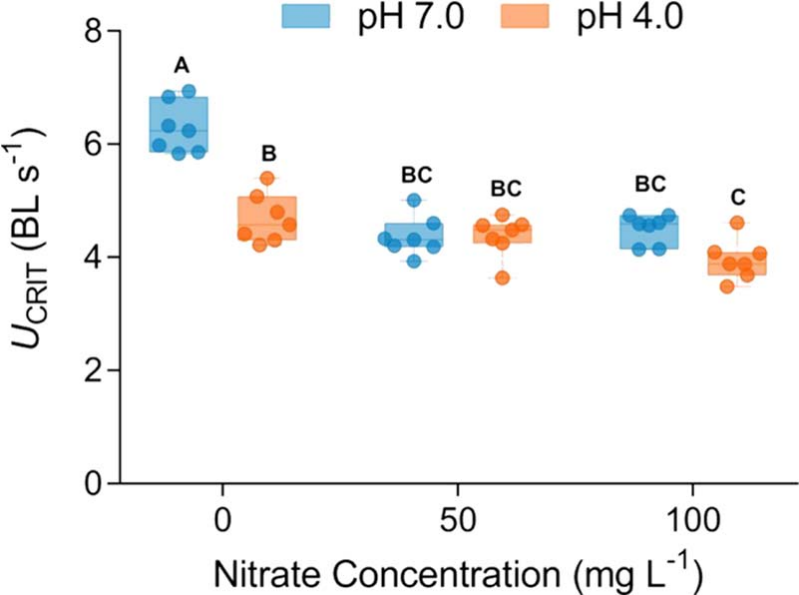
Critical swimming speed (*U*_CRIT_) of spangled perch (*L. unicolor*) exposed to a factorial combination of two pH (7.0 or 4.0) and three nitrate treatments (0, 50 or 100 mg L^−1^). Uppercase letters represent statistical difference between treatment groups. Data are presented as boxplots [minimum, first quartile (Q1), median, third quartile (Q3) and maximum], and dots represent individual data points (*n* = 7).

### Swimming performance

The *U*_CRIT_ of spangled perch was significantly affected by the interaction between nitrate and low pH (*F*_2,19_ = 11.78, *P* = 0.005), such that swimming performance was lowest in fish exposed to 100 mg L^−1^ N${\mathrm{O}}_3^{-}$ at pH 4.0 and represented a 37% reduction in *U*_CRIT_ relative to the control group (pH 7.0, Fig. 1). Independently, exposure to elevated nitrate significantly reduced the *U*_CRIT_ of fish (*F*_2,19_ = 8.14, *P =* 0.003), as did exposure to low pH 4.0 (*F*_1,19_ = 40.37; *P <* 0.001). Body length had a significant, positive influence on *U*_CRIT_ (*F*_1,19_ = 15.33, *P* < 0.0001) and was retained as a covariate in the analysis.

### Oxygen uptake and EPOC

**Figure 2:**
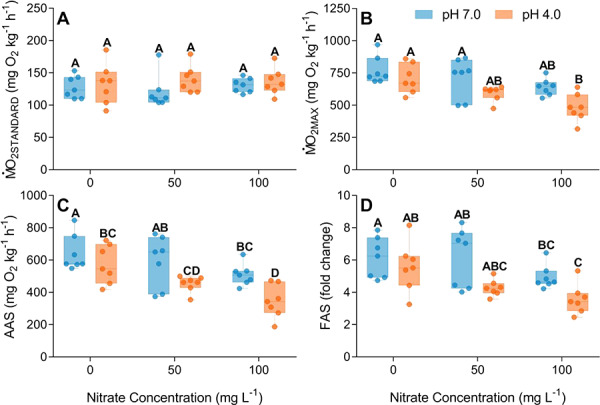
Metabolic traits (mg O_2_ kg^−1^ h^−1^) of spangled perch (*L. unicolor*) following chronic 28-day exposure to a factorial combination of two pH (7.0 or 4.0) and three nitrate treatments (0, 50 or 100 mg L^−1^). (**A**) $\dot{\mathrm{M}}$O_2STANDARD_; (**B**) Maximal oxygen uptake, $\dot{\mathrm{M}}$O_2MAX_; (**C**) AAS; (**D**) FAS. Data presented as boxplots [minimum, first quartile (Q1), median, third quartile (Q3) and maximum], and dots represent individual data points (n = 7).

Neither elevated nitrate (50 or 100 mg L^−1^${\mathrm{NO}}_3^{-}$; *F*_2,12_ = 0.27, *P* = 0.76), water pH (*F*_1_,_12_ = 0.44, *P* = 0.52) nor their interaction (*F*_2_,_12_ = 0.68, *P* = 0.52) influenced standard rates of oxygen uptake ($\dot{\mathrm{M}}$O_2STANDARD_; Fig. 2A). Conversely, increasing nitrate levels (*F*_2_,_14_ = 10.20, *P* = 0.001) and decreasing water pH (*F*_1_,_14_ = 10.29, *P* = 0.006) reduced maximum oxygen uptake ($\dot{\mathrm{M}}$O_2MAX_; Fig. 2B). However, the interaction between nitrate and pH was not significant (*F*_2_,_12_ = 0.88, *P* = 0.44), representing an additive effect of nitrate and pH on $\dot{\mathrm{M}}$O_2MAX_. On average, exposure to pH 4.0 reduced $\dot{\mathrm{M}}$O_2MAX_ by 8.5% compared with pH 7.0-exposed fish, while increasing nitrate concentration caused a stepwise decrease in $\dot{\mathrm{M}}$O_2MAX_. The largest decrease in $\dot{\mathrm{M}}$O_2MAX_ was measured in fish exposed to 100 mg L^−1^ nitrate and pH 4.0 simultaneously, which was reduced by ~47.6% relative to control (pH 7.0, 0 mg L^−1^ N${\mathrm{O}}_3^{-}$) fish. Exposure to low pH and elevated nitrate reduced both AAS and FAS in exposed fish. There was a significant main effect of pH and nitrate on AAS (pH: *F*_1,14_ = 12.16, *P* = 0.004; nitrate: *F*_2,14_ = 10.03, *P* = 0.004; Fig. 2C) and FAS (pH: *F*_1,14_ = 10.12, *P* = 0.006; nitrate: *F*_2,14_ = 5.85, *P* = 0.01; Fig. 2D), but there was no interaction between the factors (AAS: *F*_2,12_ = 0.84, *P* = 0.45; FAS: *F*_2,12_ = 1.05, *P* = 0.38) indicating an additive effect between nitrate and pH on aerobic scope.

**Figure 3:**
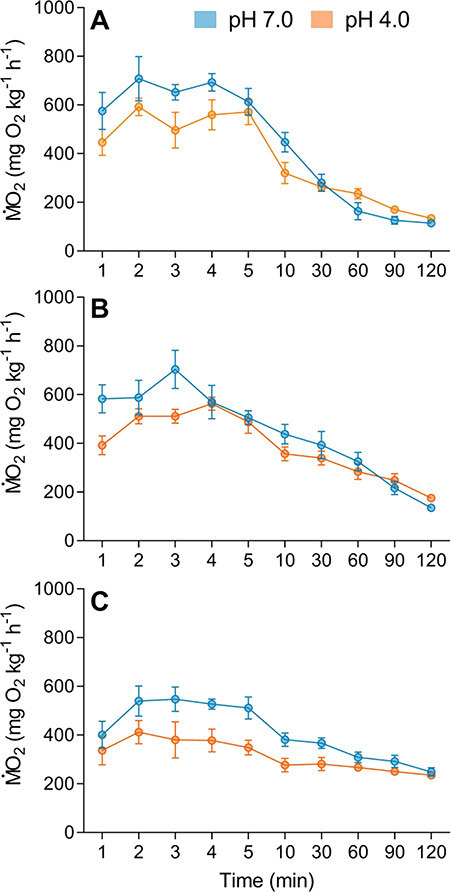
Excess post-exercise oxygen consumption (mg O_2_ kg^−1^ h^−1^) of spangled perch (*L. unicolor*) exposed to pH 7.0 (blue) or pH 4.0 (orange) and either (**A**) 0, (**B**) 50 or (**C**) 100 mg L^−1^ nitrate. Oxygen uptake was measured at 1, 2, 3, 4, 5, 10, 30, 60, 90 and 120 min post fatigue. Data presented as mean ± standard error (*n* = 7).

There was a significant effect of nitrate (*F*_*2,*14_ = 7.42, *P =* 0.006) but not pH (*F*_*1,*14_ = 2.35, *P =* 0.14; Fig. 3A) on EPOC. Exposure to nitrate doubled the amount of oxygen consumed following exercise, irrespective of nitrate concentration (Fig. 3B and C). When combined with low pH, total EPOC was also elevated compared with controls (*F*_1,12_ = 4.07, *P =* 0.04). There was a congruent effect of nitrate exposure on EPOC duration, an effect that steepened in a concentration dependent manner (50 mg L^−1^${\mathrm{NO}}_3^{-}$: *z* = 3.21, *P <* 0.001; 100 mg L^−1^${\mathrm{NO}}_3^{-}$: *z* = 3.59, *P <* 0.0001); conversely, exposure to low pH had a small but non-significant effect on EPOC duration (*z* = −1.69, *P* = 0.09). Simultaneous exposure to elevated nitrate and low pH did not enhance the duration of EPOC (pH 4.0 and 50 mg L^−1^${\mathrm{NO}}_3^{-}$: *z* = 0.80, *P =* 0.422; pH 4.0 and 100 mg L^−1^${\mathrm{NO}}_3^{-}$: *z* = 1.12, *P =* 0.22).

### Blood parameters

Plasma nitrate levels (Table 3) were significantly affected by both nitrate (*F*_2,10_ = 126.40, *P* < 0.0001) and low pH (*F*_1,10_ = 12.11, *P* < 0.01) but not by their interaction (*F*_2,8_ = 0.44, *P* = 0.65). Plasma nitrate concentrations increased linearly with increasing water–nitrate concentration in an additive fashion and were greatest in fish exposed to 100 mg L^−1^${\mathrm{NO}}_3^{-}$ at pH 4.0. Further, plasma nitrite concentrations were significantly elevated following chronic exposure to elevated water nitrate levels (*F*_2,10_ *=* 120.50; *P* < 0.0001) but were not affected by low pH (*F*_1,10_ = 0.62, *P* = 0.44). Fish body mass did not influence nitrate (*F*_1,21_ = 0.48, *P* = 0.49) or nitrite (*F*_1,21_ = 1.06, *P* = 0.31) concentrations within the plasma. Haemoglobin concentrations ([H_B_]: *F*_2,20_ = 3.56, *P* = 0.04), methaemoglobin concentrations (MetH_B_: *F*_2,20_ = 13.56, *P* = < 0.001) and haematocrit (H_CT_: *F*_2,20_ = 27.50, *P* < 0.0001), but not mean corpuscular haemoglobin concentration (MCHC: *F*_2,20_ = 0.09, *P* = 0.91), were significantly affected by exposure to elevated nitrate (Table 3). However, the effects on blood oxygen-carrying capacity were not influenced by exposure to pH 4.0 ([H_B_]: *F*_1,20_ = 0.09, *P* = 0.77; MetH_B_: *F*_1,20_ = 1.76, *P* = 0.20; H_CT_: *F*_1,20_ = 1.51, *P* = 0.23; MCHC: *F*_1,20_ = 0.64, *P* = 0.43) or by the interaction between nitrate and low pH ([H_B_]: *F*_2, 18_ = 2.63, *P* = 0.09; MetH_B_: *F*_2,18_ = 0.13, *P* = 0.88; H_CT_: *F*_2,18_ = 1.82, *P* = 0.19; MCHC: *F*_2,18_ = 1.45, *P* = 0.26) indicating an additive effect of nitrate and pH on the measured blood variables.

**Table 3:** Blood parameters of spangled perch (*L. unicolor*) following prolonged exposure to a factorial combination of two pH (7.0 or 4.0) and three nitrate treatments (0, 50 or 100 mg L^−1^)

	pH 4.0			pH 7.0		
Nitrate (mg L^−1^)	0	50	100	0	50	100
Plasma ${\mathrm{NO}}_2^{-}$	7.0 (± 0.1)^A^	90.1 (± 1.0)^B^	109.8 (± 3.7)^B^	3.0 (± 0.6)^A^	81.6 (± 16.2)^B^	98.1 (± 3.3)^B^
Plasma ${\mathrm{NO}}_3^{-}$	38.6 (± 4.5)^B^	181.8 (± 16.0)^D^	424.0 (± 46.5)^E^	4.4 (± 0.8)^A^	120.1 (± 27.6)^C^	320.8 (± 42.0)^D^
H_B_ (g dL^−1^)	12.3 (± 0.3)^A^	10.7 (± 0.3)^AB^	9.8 (± 0.5)^B^	11.1 (± 0.6)^AB^	10.5 (± 0.6)^AB^	10.9 (± 0.6)^AB^
Functional H_**B**_ (%)	94.4 (± 0.6)^A^	88.9 (± 2.0)^A^	79.4 (± 1.6)^B^	93.7 (± 0.6)^A^	88.3 (± 2.1)^A^	87.8 (± 1.6)^A^
MetHb (%)	5.6 (± 0.6)^A^	11.1 (± 2.0)^BC^	20.6 (± 1.6)^C^	6.3 (± 0.6)^AB^	11.7 (± 2.1)^ABC^	12.2 (± 1.6)^C^
Haematocrit (%)	35.4 (± 0.5)^A^	31.4 (± 0.4)^B^	30.0 (± 0.5)^B^	35.8 (± 0.9)^A^	30.7 (± 0.7)^B^	32.0 (± 0.7)^B^
MCHC (g dL^−1^)	3.5 (± 0.1)^A^	3.4 (± 0.1)^A^	3.3 (± 0.2)^A^	3.1 (± 0.1)^A^	3.4 (± 0.2)^A^	3.4 (± 0.2)^A^

Data are presented as mean ± standard error (*n* = 7 per treatment).

Superscript uppercase letters represent statistical difference between treatment groups.

### Oxygen equilibrium curves

**Figure 4:**
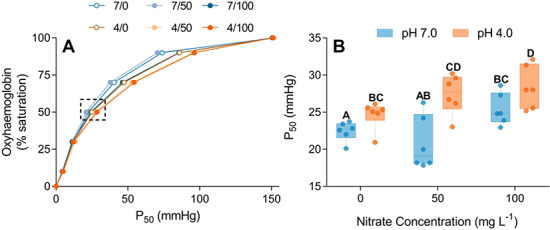
Oxygen equilibrium curves (**A**) of spangled perch (*L. unicolor*) following exposure to a factorial combination of two pH (7.0 or 4.0) and three nitrate treatments (0, 50 or 100 mg L^−1^). Graded blue lines (pH 7.0) and graded orange lines (pH 4.0) represent increasing nitrate concentrations. The dotted box in panel A highlights the point at which 50% of the haemoglobin are saturated (P_50_; mmHg), and this point is magnified in panel B. Data presented as mean ± standard error (*n* = 7).

Exposure to elevated nitrate or pH 4.0 caused a right shift in OECs (Fig. 4A). The *P*_50_ of pH 4.0-exposed fish was significantly higher *F*_1,13_ = 13.29, *P* = 0.003) than pH 7.0-exposed individuals, as was the *P*_50_ of nitrate-exposed (50 and 100 mg L^−1^) compared with control (no nitrate) fish (*F*_2,13_ = 3.82, *P* = 0.04; Fig. 4B). However, the interaction between pH and nitrate treatments on *P*_50_ values was not significant (*F*_2,11_ = 2.17, *P* = 0.16) indicating an additive effect of nitrate and low pH on *P*_50_.

## Discussion

Chronic exposure to both elevated nitrate levels and acidic pH caused numerous physiological disturbances to spangled perch. Most significantly, maximum oxygen uptake ($\dot{\mathrm{M}}$O_2MAX_) rates were reduced, and fish swimming performance and post-exercise recovery rates were impaired. Re ductions to $\dot{\mathrm{M}}$O_2MAX_ were likely a result of lowered blood oxygen-carrying capacity, since haemoglobin concentrations and haematocrit were reduced by elevated nitrate exposure, and oxygen binding affinity was compromised in fish exposed to elevated nitrate and low pH. However, contrary to our hypothesis, the interactions between nitrate and pH were predominantly additive or antagonistic and differed between physiological traits. Together, our results suggest that chronic exposure to elevated nitrate and low pH combine to exacerbate the physiological constraints placed on fish living in water of poor water quality.

### Oxygen uptake and fish functional performance

Key functional performance traits, such as locomotor capacity, growth and recovery from exhaustive exercise, are critically dependent on an organism’s capacity to take up and transport oxygen. Factors that limit oxygen uptake (pollutants, oxygen availability and ammonia) constrain active metabolism and reduce aerobic scope (Claireaux and Lefrançois, 2007; Sokolova, 2013). Prolonged exposure to elevated nitrate and acidic pH caused reductions in blood oxygen carrying, which impeded $\dot{\mathrm{M}}$O_2MAX_ capabilities and in turn reduced to the aerobic scope of juvenile spangled perch. The impact of elevated nitrate on maximal oxygen uptake was concentration-dependent and was exacerbated by simultaneous exposure to a low pH in an additive fashion. The primary cause of a reduced aerobic scope is likely the decline in blood oxygen-carrying capacity caused by nitrate and low pH exposures (as discussed below) although other mechanisms, including the impairment of gas transfer across the gills of nitrate-exposed (Schram *et al.*, 2014a; Pereira *et al.*, 2017) and low pH-exposed (McDonald *et al.*, 1991) fish, may explain the observed effect on aerobic scope. Declines in aerobic scope have been similarly documented following acute nitrite (Lefevre *et al.*, 2011) and chronic nitrate exposures (Gomez Isaza *et al.*, 2018) as well as in low-pH exposed fish (Wilson *et al.*, 1994), suggesting that elevated levels of nitrogenous waste products and altered pH regimes can have pronounced effects on the energy budget of aquatic biota.

Declines in aerobic scope coincided with a reduced swimming performance of spangled perch chronically exposed to elevated nitrate and low pH. Contrary to our predictions, simultaneous exposure to nitrate and low pH resulted in an antagonistic interaction on fish swimming performance. An antagonistic interaction refers to an effect that was less than the sum of the two stressors independently (Côté *et al.*, 2016). From the aerobic scope data, it would be expected that simultaneous exposure to elevated nitrate and low pH (which caused a 47.7% decrease in AAS) would cause a greater decrease in swimming performance than was seen. In contrast, our results indicate that prolonged swimming performance, which is partly supported by anaerobic metabolism (Plaut, 2001), is not completely governed by changes in aerobic scope. Antagonistic effects can arise following physiological compensation or due to cross-tolerance among co-occurring stressors (Sinclair *et al.,* 2013). Long-term acclimation to nitrate or low pH may have ameliorated the negative effects on swimming performance, by making compensatory changes to the cardio-respiratory system (e.g. increase heart mass/cardiac output, increase production of red-blood cells) or by increasing protective mechanisms (e.g. increase activity of protective enzymes). Antagonistic interactions can be seen as a ‘best-case scenario’, yet they represent an unfortunate situation because efforts to lessen or eliminate one stressor will not yield proportional benefits to ameliorate the observed effects on organisms (Brown *et al.* 2013; Piggott *et al.* 2015). Therefore, fish exposed simultaneously to elevated nitrate and low pH would require the alleviation of both two stressors if decrements in swimming performance were to be reversed.

We also found that the interaction between elevated nitrate and acidic pH exposures increased post-exercise recovery times such that, after 2 h, the $\dot{\mathrm{M}}$O_2_ of fish exposed to nitrate (100 mg L^−1^) and low pH was significantly elevated compared with control (unexposed) fish. Extended post-exercise recovery times likely stem from a reduced blood oxygen-carrying capacity and reflect a reduced ability to pay off an oxygen debt. Collectively, these data indicate that decrements in swimming performance are likely to pose significant fitness constraints on fish living in waters polluted by nitrate and low pH as fish need to move to evade predators, seek out resources and perform repeated locomotor activities, which may be limited by extended recovery times (Wolter and Arlinghaus, 2003; Zeng *et al.*, 2010; Zhang *et al.*, 2018).

Growth and fish condition were also compromised by elevated nitrate and low pH exposures. In fact, fish exposed to elevated nitrate and/or low pH grew <1% of their initial body mass over a 28-day period and some fish even lost mass despite being fed daily to satiety. This result has important implications for the fitness of juvenile spangled perch as growth potential is inversely related to predation risk (Post and Evans, 1989; Ribeiro and Qin, 2015) and provides a competitive advantage for resources (Cutts *et al.*, 1999). Reductions in growth have been reported following chronic nitrate exposure in several (McGurk *et al.*, 2006; van Bussel *et al.*, 2012; Lou *et al.*, 2016; Monsees *et al.*, 2017), but not all (Davidson *et al.*, 2014; Schram *et al.*, 2014a), fish species and points towards species-specific toxicity. Here, declines in aerobic scope in nitrate-exposed fish suggest that energy is diverted away from growth and is instead preferentially invested towards the detoxification and conversion of MetH_B_ back to H_B_ by the energy dependent NADH-methaemoglobin reductase enzyme (Jensen and Nielsen, 2018). Exposure to elevated nitrate concentrations can also impact on fish digestion efficiency by increasing the specific dynamic action response (SDA; i.e. the energetic costs associated with feeding and digestion) and may reduce the assimilation of feed thereby limiting growth performance (Steinberg *et al.*, 2018). Energy redistribution away from growth may also account for the poor growth performance seen in pH 4.0-exposed fish, which experienced reductions in condition factor—leading to smaller and thinner fish. The maintenance of ionic homeostasis at low pH is energetically expensive (Ye *et al.*, 1991; Wilson *et al.*, 1994) and represents a persistent physiological challenge such that growth is compromised. Therefore, while juvenile spangled perch can tolerate extreme acidic exposure, they are unlikely to thrive in acidic environments.

### Blood and oxygen-carrying capacity

Plasma nitrate concentrations tended to be higher in pH 4.0-exposed individuals, lending some support to our hypothesis that simultaneous exposure to nitrate and low pH would increase the accumulation of nitrate within the blood’s plasma. Plasma nitrate increased linearly with increasing ambient nitrate concentration; however, it remained far below environmental levels suggesting that nitrate has a low branchial permeability in spangled perch, as has been reported by previous research (Jensen, 1996; Stormer *et al.*, 1996). This result has important implications because nitrate is often regarded as relatively non-toxic due to its slow intrusion rate (Jensen, 1996); however, simultaneous exposure to other environmental stressor can exacerbate its uptake. Low pH exposure is known to increase the uptake of some pollutants directly by affecting binding sites or indirectly due to epithelial damage of the gills (Ytrestøyl *et al.*, 2001; Çoĝun and Kargin, 2004). In the case of nitrate, exposure to low pH may have compromised the integrity of the gill epithelium and facilitated the passive influx of nitrate into the blood (Jensen, 1996; Stormer *et al.*, 1996). Similar experiments on nitrite-exposed fish have reported strong haematological effects (elevated methaemoglobin, nitrite concentrations) following low pH exposure (Meade and Perrone, 1980) but others found no influence of low pH on nitrite toxicity when fish were exposed to less extreme pH condition (pH above 5 and below 10; Wedemeyer and Yasutake, 1978; Bath & Eddy, 1980). The influence of low environmental pH on nitrite/nitrate toxicity likely depends on how far below the species’ adapted pH range the fish is exposed to. Plasma nitrite concentrations were also elevated in nitrate-exposed fish. Since water nitrite concentrations tended to be negligible in all treatments (0–2 μM), the elevated levels of nitrite within the plasma are attributed to the *in vivo* conversion of nitrate to nitrite. Overall, our measures of plasma nitrite and nitrate are consistent with other accounts on various freshwater teleosts following chronic nitrate (Schram *et al.*, 2014a; Schram *et al.*, 2014b; Freitag *et al.*, 2015; Monsees *et al.*, 2017) and nitrite (Avilez *et al.*, 2004; Lefevre *et al.*, 2011) exposures and elevated levels of nitrate within the plasma are expected to cause disruptions to blood oxygen-carrying capacity of spangled perch.

Contrary to our expectations, nitrate and low pH did not combine to impede the oxygen-carrying capacity of fish. Exposure to acidic pH did not impact blood oxygen-carrying capacity, as measured by haematocrit levels, haemoglobin and methaemoglobin concentrations while nitrate exposure did influence haematological parameters. Exposure to nitrate (50 and 100 mg L^−1^) resulted in concentration-dependent effects on functional haemoglobin and methaemoglobin concentrations. Levels of haemoglobin and methaemoglobin were, however, only marginally affected (although significantly so), with the most evident effects recorded in fish exposed to 100 mg L^−1^ of nitrate and acidic pH simultaneously. Monsees *et al.* (2017) reported similar marginal effects on the blood of nitrate-exposed tilapia, whereby prolonged exposure to 500–1000 mg L^−1^${\mathrm{NO}}_3^{-}$ increased methaemoglobin and decreased haemoglobin concentrations compared with unexposed fish. Conversely, others have found no effect of nitrate exposure on blood oxygen-carrying capacity (van Bussel *et al.*, 2012; Schram *et al.*, 2014a). Interestingly, Schram *et al.*, (2014a) and van Bussel *et al.* (2012) reported an increase in plasma nitrate/nitrite concentrations of nitrate-exposed fish but no effects on blood-carrying capacity. Species-specific differences in nitrate toxicity therefore appear unrelated to a species’ capacity to maintain low plasma–nitrate concentrations. Instead, the handling of nitrate within the body (e.g. elimination, storage or detoxification capabilities) is perhaps a better indicator of nitrate toxicity. Further, we found that haematocrit levels were reduced by the presence of nitrate (from 35.8 to ~ 31%), irrespective of pH and exposure concentration. Haematocrit values of 31% are, however, within the normal range of various other tropical, freshwater species (Wells *et al.*, 1997; Witeska, 2015) and may reflect only mild nitrate-induced anaemia. Still, a decrease in haematocrit of this magnitude may be sufficient to constrain oxygen transport (Gallaugher *et al.*, 1995).

Oxygen transport capacity was restricted additively by simultaneous nitrate and low pH exposure, which caused significant right shifts in oxygen binding curves. A right shift in oxygen binding indicates reduced oxygen loading at the gills—an effect that has been previously documented in nitrite-exposed fish, including carp (*Cyprinus carpio*) (Jensen *et al.*, 1987; Jensen, 1990; Williams *et al.*, 1993) and rainbow trout (*Oncorhynchus mykiss*) (Nikinmaa and Jensen, 1992), as well as in fish exposed to other anions (Cl^−^, CO_2_, organic phosphates) (Weber and Campbell, 2010) and fish at low pH (Wood and McDonald, 1982; Ye *et al.* 1991). A decrease in oxygen binding affinity is attributed to the shrinkage of the red blood cells perhaps caused by an ionic imbalance in nitrate-exposed fish (Stormer *et al.*, 1996). Red blood cell shrinkage increases the intracellular concentrations of haemoglobin and nucleoside triphosphates thereby increasing complexing and decreasing oxygen affinity (Jensen *et al.*, 1987). On top of red blood cell shrinkage, elevated levels of nitrite in the plasma can interfere with the adrenergically activated Na*^+^*/H^+^ exchanger and inhibit the restoration of regular red blood cell volume (Nikinmaa and Jensen, 1992; Brauner and Jensen, 2002). Further to the effects of nitrate, exposure to acidic water is known to cause extracellular acidosis (Wood and McDonald, 1982), which reduces oxygen affinity via the Root effect (Root, 1931; Rummer *et al.*, 2013). Fish exposed to acidic water have limited capacity to compensate for right shifts in oxygen binding curves (Ye *et al.*, 1991) making oxygen uptake at the gills more difficult and ultimately constraining aerobic performance. Together, chronic exposure to elevated nitrate and low pH likely compounded the effects on blood-carrying capacity of spangled perch, leading to declines in fish functional performance.

## Conclusion

Freshwater species are being confronted with an increasing number of environmental challenges, thus experimental assessments of species responses to multiple threats are key in improving the management of freshwaters and their biota (McKenzie *et al.*, 2016). Water quality guidelines (Environment Australia, 2000; EPA, 2002; Canadian Council of Ministers of the Environment, 2012; European Commission, 2018) currently ignore interactions among environmental stressors, despite their complex and often exacerbated, negative effects. Here, we show that exposure to elevated nitrate posed significant ecophysiological constraints on juvenile spangled perch, and the effects are intensified by simultaneous exposure to low pH. Moreover, the effects of nitrate tended to be independent of exposure concentration (50 versus 100 mg L^−1^${\mathrm{NO}}_3^{-}$), which indicates that current nitrate water quality guidelines in Australia are unlikely to offer adequate protection for juvenile spangled perch experiencing prolonged exposures to elevated concentrations. These data highlight the importance of examining stressor interactions in light of ongoing global change and suggest that conservation and management actions need to mitigate multiple environmental threats simultaneously. Overall, our results call for a revaluation of nitrate water quality guidelines that may need to be more stringent in waterways affected by acidic effluents.
